# Association of Serum KL-6 with Functional Impairment in Patients with Immune-Mediated Interstitial Lung Disease

**DOI:** 10.3390/medicina62091697

**Published:** 2026-09-04

**Authors:** María Gema Bonilla, Carlos Carpio, Luis Gómez, Pablo Mariscal, María Torres, Luis Parra, Chamaida Plasencia-Rodríguez, María Luisa González-Casaus, María Gemma Serrano, Antonio Buño, Inmaculada Pinilla, Ana Rodríguez, Isabel Esteban, Elena Villamañan, Rodolfo Álvarez-Sala

**Affiliations:** 1 Department of Rheumatology, Hospital Universitario La Paz, 28046 Madrid, Spain; gemabonilla@ser.es (M.G.B.); chamaida.plasencia@salud.madrid.org (C.P.-R.); 2 Department of Medicine, Universidad Autónoma de Madrid, 28049 Madrid, Spain; lgomezc@salud.madrid.org (L.G.); pmaguilar91@gmail.com (P.M.); mtvargas@salud.madrid.org (M.T.); luis.pascualp@estudiante.uam.es (L.P.); elena.villamanan@salud.madrid.org (E.V.); rodolfo.alvarezsala@salud.madrid.org (R.Á.-S.); 3 Instituto de Investigación Hospital Universitario La Paz (IdiPAZ), 28046 Madrid, Spain; mgcasaus@salud.madrid.org (M.L.G.-C.); mariagemma.serrano@salud.madrid.org (M.G.S.); antonio.buno@salud.madrid.org (A.B.); 4 Department of Pulmonology, Hospital Universitario La Paz, 28046 Madrid, Spain; 5 Department of Clinical Laboratory, Hospital Universitario La Paz, 28046 Madrid, Spain; 6 Department of Radiology, Hospital Universitario La Paz, 28046 Madrid, Spain; minmaculada.pinilla@salud.madrid.org; 7 Department of Pathology, Hospital Universitario La Paz, 28046 Madrid, Spain; anamargarita.rodriguez@salud.madrid.org (A.R.); misabel.esteban@salud.madrid.org (I.E.); 8 Department of Pharmacy, Hospital Universitario La Paz, 28046 Madrid, Spain

**Keywords:** Krebs von den Lungen-6, immune-mediated interstitial lung disease, pulmonary diffusing capacity

## Abstract

*Background and Objectives:* Krebs von den Lungen-6 (KL-6) is a promising biomarker of interstitial lung disease (ILD), but data in immune-mediated ILD remain limited. We aimed to evaluate the clinical, functional, and radiological correlates of serum KL-6 levels in a real-world cohort of patients with immune-mediated ILD. *Materials and Methods:* We conducted a retrospective, cross-sectional, single-center study including adults with multidisciplinary follow-up for immune-mediated ILD and at least one serum KL-6 determination between January 2023 and September 2025. Clinical, laboratory, radiological, and pulmonary function data closest to the index KL-6 measurement were collected. KL-6 was analyzed as both a continuous variable and a categorical variable using a predefined cutoff of 500 U/mL. Correlation analyses and multivariable logistic regression were performed to identify factors independently associated with elevated KL-6 levels. *Results:* A total of 112 patients were included; 72.3% were women, with a median age of 70 years (IQR 63–77). The most frequent underlying diseases were systemic sclerosis (30.4%), rheumatoid arthritis (25.9%), and inflammatory myopathy (14.3%). Median KL-6 concentration was 770.5 U/mL (IQR 437.3–1148.5). Patients with FVC < 80% predicted and DLCO < 60% predicted had significantly higher KL-6 levels than those with better lung function (*p* = 0.001 and *p* = 0.004, respectively). KL-6 levels correlated inversely with DLCO (% predicted) (ρ = −0.388, *p* < 0.001) and FVC (% predicted) (ρ = −0.328, *p* < 0.001) but not with the presence of radiological fibrosis. In the multivariable analysis, lower DLCO (% predicted) (OR 0.919, 95% CI 0.884–0.955; *p* < 0.001) and older age (OR 1.063, 95% CI 1.014–1.115; *p* = 0.012) were independently associated with elevated KL-6 levels. *Conclusions:* Elevated serum KL-6 concentrations were associated with impaired gas transfer as measured by DLCO % predicted in this cross-sectional cohort. Longitudinal studies are required to determine whether KL-6 can predict functional decline, disease progression, or clinical outcomes.

## 1. Introduction

Interstitial lung disease (ILD) is a frequent and clinically significant manifestation of immune-mediated diseases, including systemic sclerosis, rheumatoid arthritis, and idiopathic inflammatory myopathies [1]. Across these conditions, ILD is associated with substantial morbidity, progressive respiratory impairment, and increased mortality, representing one of the major determinants of long-term outcomes. Despite advances in diagnosis and treatment, disease severity and progression remain highly variable and often difficult to predict [2,3].

The assessment of ILD currently relies on high-resolution computed tomography (HRCT) and pulmonary function tests (PFTs), particularly forced vital capacity (FVC) and diffusing capacity of the lung for carbon monoxide (DLCO) [1,4]. However, these tools may not fully capture ongoing alveolar epithelial injury and may be limited for frequent monitoring [2]. Therefore, there is growing interest in identifying circulating biomarkers that can provide complementary information regarding disease severity and pulmonary involvement [2,5]. Krebs von den Lungen-6 (KL-6), a high-molecular-weight glycoprotein expressed by type II alveolar epithelial cells and released into the circulation in association with epithelial damage and regeneration, has emerged as one of the most extensively investigated and clinically promising biomarkers in ILD [6,7]. Elevated serum KL-6 levels have been associated with the presence, extent, and progression of lung disease in several ILD subtypes [8].

Most studies evaluating KL-6 have focused on specific autoimmune diseases, particularly systemic sclerosis, rheumatoid arthritis and idiopathic inflammatory myopathies [9,10,11]. More recently, KL-6 has been further recognized as a potentially valuable biomarker in connective tissue disease-associated ILD, with studies demonstrating associations with the presence of ILD, pulmonary function impairment, and disease severity across different autoimmune conditions [12]. Emerging evidence also suggests that combining KL-6 with non-invasive imaging modalities, such as lung ultrasound, may improve the identification and assessment of pulmonary involvement [13]. However, most available evidence derives from disease-specific or selected research cohorts, and whether KL-6 consistently reflects ILD severity across a broad spectrum of immune-mediated diseases in routine clinical practice remains insufficiently characterized. In particular, its relationship with established indicators of severity, including pulmonary function impairment and radiological abnormalities, requires further evaluation in heterogeneous real-world populations. The potential role of KL-6 in monitoring the response to antifibrotic treatment remains uncertain. A recent systematic review and meta-analysis evaluating serial KL-6 concentrations in patients with ILD receiving nintedanib or other antifibrotic therapy found no statistically significant pooled change in KL-6 levels during treatment, although substantial heterogeneity was observed across studies. These findings suggest that KL-6 concentrations may remain relatively stable during antifibrotic therapy, but larger and methodologically standardized longitudinal studies are required to determine whether serial KL-6 measurement can provide clinically meaningful information regarding treatment response [14].

Therefore, this study aimed to examine the relationship between serum KL-6 concentrations and pulmonary function, radiological features, and antifibrotic treatment in a real-world cohort of patients with immune-mediated ILD.

## 2. Materials and Methods

### 2.1. Study Design and Population

**Figure 1:**
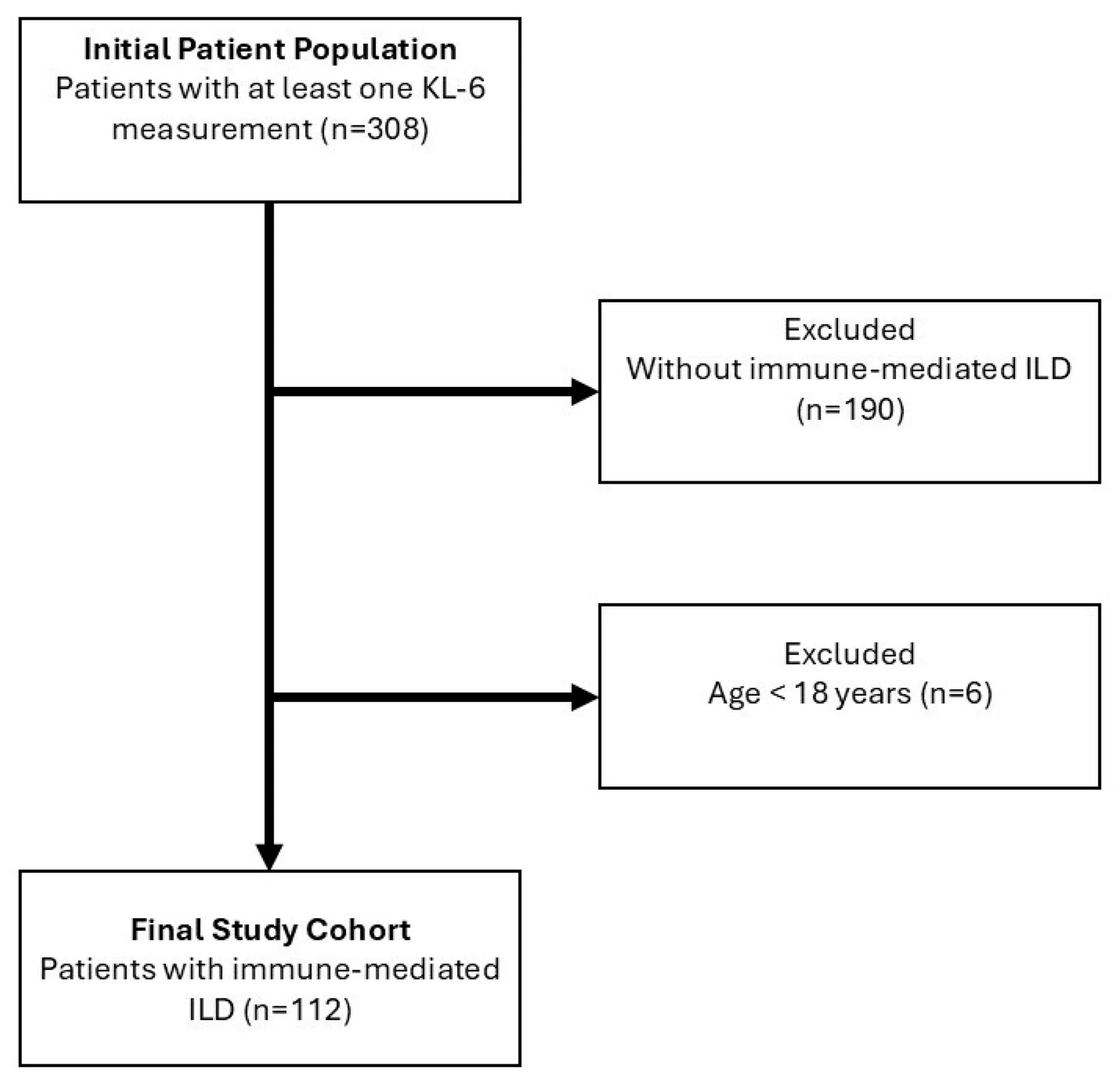
Patient selection flowchart. The flowchart illustrates patient selection and the exclusion criteria applied to derive the final cohort of 112 patients with immune-mediated interstitial lung disease. ILD, interstitial lung disease; KL-6, Krebs von den Lungen-6.

This retrospective, cross-sectional, single-center observational study was conducted at the multidisciplinary clinic for immune-mediated diseases and interstitial lung disease (ILD), jointly managed by the Departments of Rheumatology and Pulmonology at La Paz University Hospital (Madrid, Spain). Adult patients (≥18 years) with a diagnosis of immune-mediated ILD who were followed at the multidisciplinary clinic and had at least one serum KL-6 measurement between January 2023 and September 2025 were eligible for inclusion. Potential participants were identified through the hospital laboratory database, which was queried for all serum KL-6 determinations performed during the study period. Patients who were not undergoing multidisciplinary follow-up for immune-mediated ILD were excluded. The diagnosis of immune-mediated ILD was based on an integrated assessment of clinical features, laboratory findings, pulmonary function tests, and HRCT reports provided by radiologists specializing in thoracic and interstitial lung diseases. These findings were interpreted by the treating pulmonologists and rheumatologists. Histopathological findings were also considered when lung biopsy had been performed. Cases with diagnostic uncertainty were discussed by a multidisciplinary committee comprising pulmonologists, rheumatologists, thoracic radiologists, and pathologists. Concomitant COPD, emphysema, or a history of malignancy were not predefined exclusion criteria, provided that patients fulfilled the eligibility criteria for immune-mediated ILD and were undergoing follow-up in the multidisciplinary clinic. The presence and severity of COPD or emphysema, pulmonary hypertension, active respiratory infection, malignancy, renal dysfunction, supplemental oxygen requirements, and standardized measures of immune-mediated disease activity or severity were not systematically collected in the study database and could therefore not be included as covariates in the adjusted analyses. After application of the eligibility criteria, 112 patients were included in the final analysis (Figure 1). To ensure that each patient contributed only one observation, the most recent available serum KL-6 measurement during the study period was selected as the index determination. Clinical, functional, radiological, and treatment-related variables were assessed in relation to this index date.

### 2.2. Clinical, Functional, and Radiological Assessment

Clinical, laboratory, radiological, and pulmonary function data were retrospectively extracted from electronic medical records. Pulmonary function tests were generally performed on the same day as the index KL-6 measurement as part of the multidisciplinary clinical assessment. When same-day pulmonary function measurements were not available, the assessment closest in time to the index KL-6 determination was selected within a predefined maximum interval of 30 days. This pragmatic window was chosen to preserve temporal proximity while minimizing missing functional data in this retrospective real-world cohort. For high-resolution computed tomography (HRCT), the closest available examination was recorded. Data unavailable within the predefined timeframe were considered missing.

Collected variables included demographic characteristics, smoking status, underlying immune-mediated disease, disease duration, respiratory symptoms, and ILD treatment. Laboratory variables included serum KL-6 concentrations, C-reactive protein (CRP), creatine phosphokinase (CPK), and disease-specific autoantibodies when available. Pulmonary function tests were performed according to current American Thoracic Society/European Respiratory Society (ATS/ERS) recommendations [15]. Spirometric parameters included forced vital capacity (FVC), forced expiratory volume in one second (FEV_1_), and the FEV_1_/FVC ratio. Hemoglobin-corrected diffusing capacity of the lung for carbon monoxide (DLCO) was also recorded. Percent predicted values were calculated using the appropriate Global Lung Function Initiative (GLI) reference equations and corresponding z-scores were obtained [15,16,17]. For comparative analyses, pulmonary impairment was categorized using predefined thresholds of FVC < 80% predicted and DLCO < 60% predicted [18].

For the purposes of this study, HRCT findings were classified as fibrotic or non-fibrotic ILD on the basis of the original reports issued by radiologists specializing in thoracic and interstitial lung diseases. Fibrotic ILD was defined by the presence of reticulation associated with traction bronchiectasis or bronchiolectasis, architectural distortion, and/or honeycombing [19,20].

### 2.3. KL-6 Measurement

KL-6 concentrations were measured in the Department of Clinical Analytics at La Paz University Hospital using the Lumipulse^®^ G KL-6 assay (Fujirebio, Tokyo, Japan) on the LUMIPULSE G automated platform. The assay is based on chemiluminescent enzyme immunoassay (CLEIA) technology. Serum KL-6 measurements were performed as part of routine clinical care at the discretion of the multidisciplinary team. No blood samples were obtained specifically for research purposes. A standardized laboratory protocol and the same analytical platform were used throughout the study period. KL-6 concentrations were analyzed as a continuous variable and were also dichotomized using a prespecified cutoff of 500 U/mL. This literature-based threshold [21] was not derived from the present dataset and was not considered a diagnostic cutoff optimized for this cohort. Because the interpretation of absolute KL-6 concentrations may vary according to the assay and clinical population, analyses based on the dichotomized variable were interpreted together with those treating KL-6 as a continuous measure.

### 2.4. Ethics Statement

This study was approved by the Research Ethics Committee (CEIm) of Hospital Universitario La Paz (approval code PI–2025.836). The study was conducted in accordance with the ethical principles of the Declaration of Helsinki and applicable national regulations.

### 2.5. Statistical Analysis

Continuous variables are presented as median and interquartile range [IQR], and categorical variables as absolute frequencies and percentages. For categorical variables with missing observations, data are reported as *n*/*N* (%), where *N* represents the number of patients with available data, and percentages were calculated within each KL-6 category. Missing values were not imputed. Descriptive and univariable analyses were conducted using available cases for each variable, whereas the multivariable logistic regression was performed as a complete-case analysis including only patients with available data for the outcome and all predictors entered into the model. Variable-specific denominators are reported in the corresponding tables.

Data distributions were assessed by graphical inspection and the Kolmogorov–Smirnov test. As several continuous variables, including serum KL-6 concentrations, showed non-normal distributions, non-parametric methods were preferentially used. Comparisons between two independent groups were performed using Student’s *t*-test or the Mann–Whitney U test, as appropriate. Associations between categorical variables were assessed using the chi-square test or Fisher’s exact test, as appropriate. Correlations between continuous variables were evaluated using Spearman’s rank correlation coefficient.

To investigate factors associated with elevated serum KL-6 concentrations, KL-6 was dichotomized using the prespecified threshold of 500 U/mL, and binary logistic regression analysis was performed with KL-6 ≥ 500 U/mL as the dependent variable. Given the limited number of patients in the smaller outcome category, the revised primary multivariable model was restricted to two predictors to reduce the risk of overfitting. Age was retained based on its clinical relevance and its association with elevated KL-6 concentrations, whereas DLCO % predicted was selected as the principal measure of pulmonary functional impairment based on its clinical relevance and the strength of its univariable association with elevated KL-6 concentrations.

FVC % predicted and DLCO % predicted were not entered simultaneously into the primary model to limit the number of predictors, preserve model parsimony, and reduce redundancy between related measures of pulmonary function. Their association was assessed using Spearman’s rank correlation coefficient. Because FVC % predicted was not entered into the final model, collinearity diagnostics were performed only for the predictors retained in the model. Antifibrotic treatment was excluded from the revised multivariable model because its markedly imbalanced distribution across the outcome categories resulted in a risk of near-complete separation and potentially unstable maximum-likelihood estimates.

Collinearity diagnostics for the final logistic regression model were obtained using an auxiliary linear regression model containing the same predictors. Multicollinearity was evaluated using tolerance and variance inflation factor values. Internal stability of the regression coefficients was assessed using 1000 bootstrap samples. Bias-corrected and accelerated 95% confidence intervals and two-sided bootstrap *p*-values were obtained for the regression coefficients.

To explore potential confounding by the underlying immune-mediated disease without overparameterizing the primary model, sensitivity analyses were performed by separately adding indicator variables for each of the three most frequent diagnostic categories, namely systemic sclerosis, rheumatoid arthritis, and inflammatory myopathy, to the model containing age and DLCO % predicted. Each diagnostic variable was coded dichotomously, comparing patients with the specified diagnosis with all other patients. These analyses were considered exploratory and were used to assess the stability of the estimates for age and DLCO % predicted across the principal diagnostic groups.

Logistic regression results are reported as odds ratios (ORs) with 95% confidence intervals (CIs). Apparent model fit was descriptively summarized using Nagelkerke’s R^2^. All statistical analyses were performed using IBM SPSS Statistics version 29 (IBM Corp., Armonk, NY, USA). All tests were two-sided, and *p*-values < 0.05 were considered statistically significant.

## 3. Results

### 3.1. Baseline Characteristics

The study included 112 patients, of whom 72.3% were women. The median age was 70 years [IQR, 63–77], and the median body mass index (BMI) was 25.6 kg/m^2^ [IQR, 23.6–28.4]. The underlying diseases were heterogeneous, mainly comprising systemic sclerosis (30.4%), rheumatoid arthritis (25.9%), and inflammatory myopathy (14.3%). Dyspnea was present in 62.2% of patients. Pulmonary function measurements within the prespecified 30-day window were available for 111 patients for FVC and 109 patients for DLCO, and it showed a median FVC of 87% predicted [73–96] and a median DLCO of 64% predicted [51.5–76.5]. Median serum KL-6 concentration was 770.5 U/mL [437.3–1148.5] (Table 1).

### 3.2. Association of KL-6 with Clinical and Functional Variables

KL-6 concentrations were significantly higher in patients with impaired pulmonary function. Patients with an FVC < 80% predicted had higher KL-6 levels than those with preserved lung function (1036 [591.5–1391.5] vs. 690 [368.5–916] U/mL; *p* = 0.001). Similarly, patients with a DLCO < 60% predicted showed higher KL-6 concentrations than those with better diffusion capacity (830 [574–1391.5] vs. 612.5 [333.8–916] U/mL; *p* = 0.004) (Table 2).

Patients with elevated KL-6 levels (≥500 U/mL) were older (*p* = 0.029), had a higher body mass index (*p* = 0.033), and more frequently reported dyspnea (*p* = 0.004) than those with lower KL-6 concentrations. Elevated KL-6 levels were also associated with lower FVC (% predicted), reduced DLCO (% predicted), and a higher FEV_1_/FVC ratio (Table 3).

KL-6 levels were higher among patients receiving antifibrotic treatment than those not receiving antifibrotic therapy (847.5 [740–1652.3] vs. 710 [370.5–1054.8] U/mL; *p* = 0.011). In contrast, no significant differences were observed according to sex, smoking status, underlying immune-mediated disease, or the presence of fibrotic changes on HRCT (*p* = 0.724) (Table 2 and Table 3).

### 3.3. Correlation Analysis

**Figure 2:**
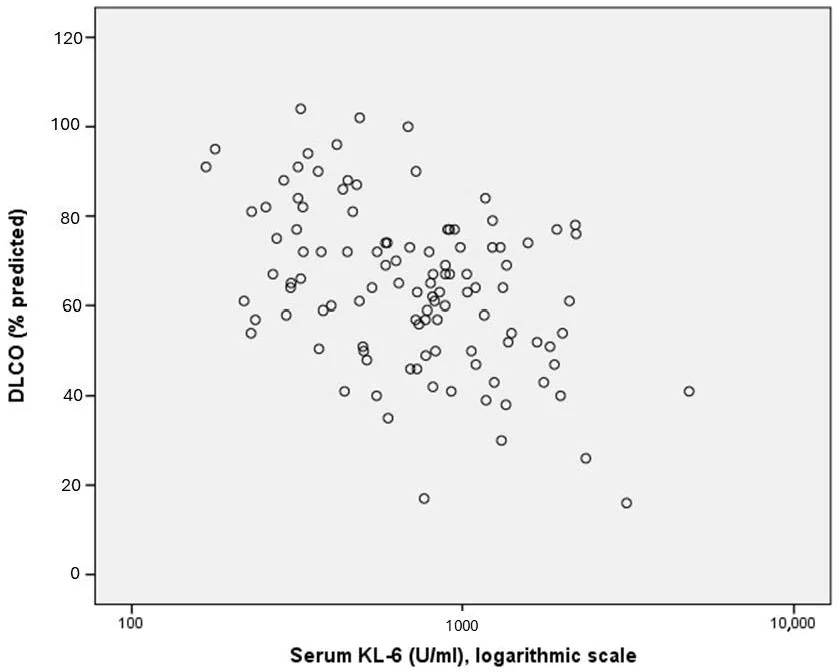
Correlation between serum KL-6 concentrations and DLCO (% predicted). Serum KL-6 concentrations are displayed on a logarithmic scale because of their right-skewed distribution. The association was assessed using Spearman’s rank correlation coefficient (ρ = −0.388, *p* < 0.001). KL-6, Krebs von den Lungen-6; DLCO, hemoglobin-corrected diffusing capacity of the lung for carbon monoxide.

KL-6 levels were inversely correlated with pulmonary function, particularly DLCO (% predicted) (ρ = −0.388, *p* < 0.001) and FVC (% predicted) (ρ = −0.328, *p* < 0.001), indicating higher KL-6 concentrations in patients with greater functional impairment (Figure 2). A moderate positive correlation was observed with the FEV_1_/FVC ratio (ρ = 0.348, *p* < 0.001). Additionally, weak correlations were identified with BMI (ρ = 0.200, *p* = 0.034) and disease duration (ρ = −0.220, *p* = 0.022), whereas no significant associations were found with CRP or CPK. Moreover, no significant difference was observed according to the presence of a fibrotic ILD pattern on HRCT.

### 3.4. Multivariable Logistic Regression Analysis

In unadjusted analyses, dyspnea (*p* = 0.004), antifibrotic treatment (*p* = 0.005), FVC % predicted (*p* = 0.013), and DLCO % predicted (*p* < 0.001) were associated with KL-6 concentrations ≥ 500 U/mL. Given the limited number of patients in the smaller outcome category, the revised primary multivariable logistic regression model was restricted to age and DLCO % predicted. The primary multivariable analysis included 107 patients with complete data for the variables entered into the model. Increasing age was independently associated with higher odds of KL-6 concentrations ≥ 500 U/mL (adjusted OR per year, 1.063; 95% CI, 1.014–1.115; *p* = 0.012), whereas increasing DLCO % predicted was associated with lower odds of elevated KL-6 (adjusted OR per one-percentage-point increase, 0.919; 95% CI, 0.884–0.955; *p* < 0.001). Thus, each one-percentage-point increase in DLCO % predicted was associated with an 8.1% reduction in the odds of KL-6 concentrations ≥ 500 U/mL.

No problematic multicollinearity was identified between age and DLCO % predicted, with tolerance and variance inflation factor values of 1.000 for both predictors. The internal stability of the regression coefficients was supported by bootstrap analysis based on 1000 samples. The bias-corrected and accelerated 95% confidence intervals for the adjusted ORs were 1.002–1.147 for age (bootstrap *p* = 0.016) and 0.869–0.944 for DLCO % predicted (bootstrap *p* = 0.002). The final model had a Nagelkerke R^2^ of 0.401. The Hosmer–Lemeshow test did not indicate evidence of lack of fit: χ^2^ = 10,622, df = 8, *p* = 0.244.

The associations of age and DLCO % predicted remained stable in exploratory sensitivity analyses that additionally included systemic sclerosis, rheumatoid arthritis, or inflammatory myopathy separately. Across these analyses, the adjusted OR for age ranged from 1.061 to 1.064, whereas that for DLCO % predicted ranged from 0.915 to 0.918. None of the three diagnostic variables was statistically significant, although their confidence intervals were wide. These analyses were not powered to compare disease-specific effects or assess interaction by underlying diagnosis.

## 4. Discussion

In this real-world cohort of patients with immune-mediated interstitial lung disease, higher serum KL-6 concentrations were cross-sectionally associated with lower pulmonary function parameters, particularly reduced DLCO. In the multivariable analysis, lower DLCO % predicted was independently associated with a greater likelihood of elevated KL-6 concentrations, supporting a cross-sectional relationship between KL-6 and impaired gas transfer.

A key finding of this study was the independent association between KL-6 levels and DLCO. Although KL-6 concentrations were associated with several pulmonary function parameters in the univariable analyses, DLCO was the only functional variable that remained independently associated with elevated KL-6 concentrations in the multivariable model. Specifically, lower DLCO % predicted was associated with a greater likelihood of presenting KL-6 concentrations ≥ 500 U/mL. This inverse association should be interpreted according to the direction of the regression model, in which elevated KL-6 was the dependent variable. Therefore, our findings do not demonstrate that KL-6 can identify patients with functional impairment or predict subsequent functional decline. Nevertheless, the observed association is biologically plausible, as DLCO reflects gas transfer across the alveolar–capillary membrane and is particularly sensitive to early and clinically relevant pulmonary involvement in ILD [22]. However, DLCO is not specific to ILD severity and may also be reduced by pulmonary hypertension, emphysema, anemia, and other pulmonary vascular or parenchymal conditions. Although DLCO values were corrected for hemoglobin, the potential effects of these additional factors could not be fully addressed in the present analysis. Because KL-6 is released by injured and regenerating type II alveolar epithelial cells, increased circulating levels are thought to reflect ongoing epithelial damage and disruption of the alveolar–capillary barrier, processes that directly impair gas exchange [23]. Our findings are consistent with previous studies in immune-mediated ILD showing significant associations between KL-6 concentrations and impaired pulmonary function, particularly reduced DLCO and more extensive lung involvement [2,24]. Moreover, elevated KL-6 levels have been associated with subsequent DLCO decline in systemic sclerosis-associated ILD [25]. Taken together, these findings support a close cross-sectional relationship between elevated KL-6 concentrations and impaired gas transfer, while the potential ability of KL-6 to identify or predict functional deterioration requires evaluation in appropriately designed longitudinal studies. The positive association observed between KL-6 concentrations and the FEV_1_/FVC ratio likely reflects the relative preservation of or increase in this ratio in restrictive ventilatory impairment, resulting from a proportionally greater reduction in FVC than in FEV_1_, rather than an independent relationship between KL-6 and the FEV_1_/FVC ratio.

Our cohort encompassed a heterogeneous spectrum of immune-mediated diseases, predominantly systemic sclerosis, rheumatoid arthritis, and inflammatory myopathy, together with smaller groups of patients with interstitial pneumonia with autoimmune features, overlap syndrome, Sjögren’s disease, sarcoidosis, and other diagnoses. In the overall cohort, higher KL-6 concentrations were associated with lower pulmonary function parameters, particularly lower DLCO. Exploratory sensitivity analyses separately accounting for the three most frequent underlying diseases did not materially alter the association between DLCO % predicted and elevated KL-6 concentrations. However, these analyses were not designed to demonstrate consistency across diseases, and the limited sample size of the individual diagnostic groups precluded adequately powered stratified analyses or formal interaction testing. Therefore, the magnitude and clinical significance of the relationship between KL-6 and pulmonary function may differ according to the underlying immune-mediated disease. Previous studies have reported associations between KL-6 and pulmonary involvement in systemic sclerosis, rheumatoid arthritis, inflammatory myopathies, and mixed immune-mediated ILD cohorts [9,10,11,12,13,24]. Nevertheless, larger disease-specific studies are required to determine whether KL-6 has comparable clinical significance across these populations.

No statistically significant difference in KL-6 concentrations was observed according to the presence or absence of fibrotic abnormalities on HRCT. However, this finding should be interpreted cautiously because HRCT involvement was classified only as fibrotic or non-fibrotic, without quantitative or semiquantitative assessment of disease extent. Therefore, the present analysis cannot determine whether KL-6 concentrations are associated with radiological fibrosis burden. KL-6 may provide biological information complementary to imaging, but this possibility requires confirmation in studies incorporating standardized quantitative or semiquantitative HRCT assessment. Our results differ from previous studies reporting positive associations between serum KL-6 concentrations and the extent of fibrotic abnormalities on HRCT. For example, Lee et al. [26] observed progressively higher KL-6 levels with increasing CT grades of lung involvement in immune-mediated ILD, while a recent study in treatment-naïve idiopathic pulmonary fibrosis demonstrated a strong correlation between KL-6 and HRCT fibrosis scores [27]. Several factors may explain these discrepancies. First, unlike previous studies that quantified the extent of fibrosis, our analysis evaluated only the presence or absence of fibrotic abnormalities, which may have limited the ability to detect associations with disease burden. Therefore, subtle relationships between KL-6 levels and the extent of radiological fibrosis may have remained undetected. Second, our cohort included a heterogeneous spectrum of immune-mediated ILDs, whereas many earlier studies focused on more homogeneous populations. Finally, the stronger association observed between KL-6 and DLCO, including its independent association in the multivariable analysis, suggests that KL-6 may be more closely linked to alveolar–capillary dysfunction and impaired gas exchange than to static radiological manifestations of fibrosis. Taken together, these findings suggest that KL-6 may provide information related to pulmonary functional impairment that is complementary to the categorical assessment of fibrotic abnormalities on HRCT.

Patients receiving antifibrotic treatment had higher serum KL-6 concentrations than those not receiving antifibrotic therapy. However, this cross-sectional association should be interpreted cautiously and should not be regarded as evidence of a treatment effect. Antifibrotic agents are generally prescribed to patients with progressive fibrotic disease or greater clinical concern; therefore, the observed difference may primarily reflect confounding by indication. Moreover, a recent systematic review and meta-analysis found no statistically significant pooled change in KL-6 concentrations following nintedanib treatment or antifibrotic therapy overall, although substantial heterogeneity was observed across studies [14]. Accordingly, our findings do not establish whether antifibrotic treatment influences KL-6 concentrations or whether serial KL-6 measurement is useful for monitoring treatment response. Prospective longitudinal studies assessing KL-6 trajectories alongside pulmonary function and relevant clinical outcomes are required to clarify this potential role.

From a practical perspective, serum KL-6 measurement may provide complementary information in the assessment of immune-mediated ILD. Although HRCT and pulmonary function testing remain the cornerstones of disease evaluation, the observed cross-sectional association between higher KL-6 concentrations and lower pulmonary function suggests that KL-6 may complement functional assessment. However, the present study did not evaluate the ability of KL-6 to identify or classify patients according to the severity of pulmonary involvement, predict disease progression, or guide clinical decision-making. Prospective longitudinal studies are required to determine its potential role in disease monitoring and to establish clinically meaningful, assay-specific thresholds.

The strengths of this study include the evaluation of a well-characterized real-world cohort encompassing a broad spectrum of immune-mediated diseases. However, this clinical heterogeneity also limits disease-specific interpretation of the findings. Nevertheless, several limitations should be acknowledged. First, the retrospective, cross-sectional design precludes causal, predictive, or prognostic interpretations. Moreover, because elevated KL-6 was modeled as the dependent variable, the multivariable analysis identifies factors associated with KL-6 concentrations ≥ 500 U/mL but does not evaluate the ability of KL-6 to detect functional impairment, classify disease severity, or predict subsequent outcomes. Second, KL-6 testing was performed at the discretion of the multidisciplinary team rather than systematically in all patients with immune-mediated ILD. This may have introduced selection or indication bias and limited the generalizability of the findings. A comparison between tested and non-tested patients could not be performed because complete data were unavailable for the latter group. Third, the heterogeneity of the underlying diseases and treatments may have influenced KL-6 concentrations and limited disease-specific interpretation. Although exploratory sensitivity analyses were performed for the three most frequent diagnoses, the study was not sufficiently powered for disease-stratified analyses or formal interaction testing. Therefore, consistency of the association across immune-mediated diseases cannot be established from the present data. The association between antifibrotic treatment and elevated KL-6 concentrations is also susceptible to confounding by indication, as discussed above. Fourth, HRCT findings were classified only as fibrotic or non-fibrotic, without quantitative or semiquantitative assessment of disease extent. Therefore, the absence of a statistically significant association between KL-6 and the presence of radiological fibrosis should be considered inconclusive. Fifth, the literature-based threshold of 500 U/mL may not fully account for assay-specific or population-specific differences in KL-6 concentrations. Finally, some variables had missing data, which were not imputed, and the analyses were based on available cases or complete cases, as appropriate. In addition, the number and timing of serial KL-6 determinations per patient were not systematically recorded, and only the last available KL-6 measurement during the study period was analyzed. Therefore, within-patient longitudinal changes in KL-6 concentrations could not be evaluated. Prospective longitudinal studies incorporating serial KL-6 measurements, pulmonary function trajectories, standardized quantification of radiological involvement, relevant comorbidities, and clinical outcomes are needed to address these limitations.

## 5. Conclusions

In this real-world cohort of patients with immune-mediated interstitial lung disease, higher serum KL-6 concentrations were cross-sectionally associated with lower pulmonary function, particularly impaired gas transfer as measured by DLCO % predicted. However, the absence of a statistically significant association with radiological fibrosis should be considered inconclusive because fibrosis was assessed using a binary classification without quantification of its extent. These findings support an association between serum KL-6 concentrations and pulmonary functional impairment at a single time point but do not establish the ability of KL-6 to classify patients according to disease severity, predict disease progression, or provide prognostic stratification. Prospective longitudinal studies incorporating serial KL-6 measurements, pulmonary function trajectories, quantitative assessment of fibrosis, and clinical outcomes are needed to clarify its potential role in disease monitoring.

## Figures and Tables

**Table 1:** Baseline characteristics of the study population.

		Available *n*	Patients (*n* = 112)
Female sex		112	81 (72.3%)
Age, years		110	70 [63–77]
BMI, kg/m^2^		111	25.6 [23.6–28.4]
Current/former smoking history		112	49 (43.8%)
Cough		111	17 (15.3%)
Dyspnea		111	69 (62.2%)
mMRC dyspnea score		111	1 (0–1)
Underlying immune-mediated disease			
	Systemic sclerosis	112	34 (30.4%)
	Rheumatoid arthritis	112	29 (25.9%)
	Inflammatory myopathy	112	16 (14.3%)
	IPAF	112	6 (5.4%)
	Overlap syndrome	112	6 (5.4%)
	Sjögren’s disease	112	6 (5.4%)
	Sarcoidosis	112	5 (4.5%)
	Other diagnoses	112	10 (8.9%)
Duration of immune-mediated disease, years		108	8 [3–15.8]
Duration of ILD, years		106	5 [2–9]
Treatment			
	Corticosteroids	112	55 (49.1%)
	Mycophenolate/Azathioprine	112	38 (33.9%)
	Rituximab	112	18 (16.1%)
	Tocilizumab	112	2 (1.8%)
	Abatacept	112	8 (7.1%)
	Leflunomide	112	11 (9.8%)
	Hydroxychloroquine	112	12 (10.7%)
	Methotrexate	112	10 (8.9%)
	Adalimumab	112	1 (0.9%)
	Baricitinib	112	1 (0.9%)
Antifibrotic treatment		112	20 (17.9%)
	Nintedanib	20	14 (70%)
	Pirfenidone	20	6 (30%)
Autoantibodies			
	ANA positive	108	75 (69.4%)
	Anti-CCP positive	77	20 (26%)
	Anti-Scl70 positive	87	18 (20.7%)
	Anti-Ro positive	86	18 (20.9%)
	Anticentromere positive	75	9 (12%)
	Rheumatoid factor, IU/mL	103	10 [8.5–36.7]
Systemic inflammatory biomarkers			
	CPK, U/L	91	74 [55–110]
	CRP, mg/L	110	3.3 [1.2–9.2]
HRCT pattern		106	
	Fibrotic ILD pattern		62/106 (58.5%)
	Non-fibrotic ILD pattern		44/106 (41.5%)
Pulmonary function tests			
	FVC, L	111	2.46 [2.10–2.96]
	FVC, % predicted	111	87 [76–96]
	FVC z-score	108	−0.74 [−1.77–−0.27]
	FEV_1_, L	111	1.92 [1.64–2.32]
	FEV_1_, % predicted	111	87 [76–97]
	FEV_1_ z-score	108	−0.77 [−1.46–−0.24]
	FEV_1_/FVC ratio	111	0.80 [0.75–0.84]
	DLCO, % predicted	109	64 [51.5–76.5]
	DLCO z-score	109	−2.09 [−2.87–−1.33]
KL-6, U/mL		112	770.5 [437.3–1148.5]

Data are presented as *n* (%) for categorical variables or median [interquartile range] for continuous variables. The “Available *n*” column indicates the number of patients with non-missing data for each variable. Percentages were calculated using the number of patients with available data as the denominator. FVC measurements obtained within 30 days of the index KL-6 determination were available for 111 patients, and DLCO measurements were available for 109 patients. Missing data were not imputed, and analyses were performed using available cases. For autoantibody variables, tests not performed or without an available result were considered missing and were not classified as negative. For treatment variables, documented absence of treatment was coded as “no” and was not considered missing. Treatment variables indicate the number and percentage of patients receiving each medication. ANA, antinuclear antibodies; anti-CCP, anti–cyclic citrullinated peptide; anti-Ro, anti–Ro/SSA antibodies; anti-Scl70, anti–topoisomerase I; BMI, body mass index; CPK, creatine phosphokinase; CRP, C-reactive protein; DLCO, hemoglobin-corrected diffusing capacity of the lung for carbon monoxide; FEV_1_, forced expiratory volume in 1 s; FVC, forced vital capacity; HRCT, high-resolution computed tomography; ILD, interstitial lung disease; IPAF, interstitial pneumonia with autoimmune features; KL-6, Krebs von den Lungen-6; mMRC, modified Medical Research Council.

**Table 2:** Comparison of serum KL-6 levels across clinical subgroups.

Subgroup	*n*	KL-6 (U/mL) Median [IQR]	*p*
Antifibrotic treatment	20	847.5 [740–1652.3]	0.011
Non-antifibrotic treatment	92	710 [370.5–1054.8]	
FVC ≥ 80% predicted	74	690 [368.5–916]	0.001
FVC < 80% predicted	37	1036 [591.5–1391.5]	
DLCO ≥ 60% predicted	68	612.5 [333.8–916]	0.004
DLCO < 60% predicted	41	830 [574–1391.5]	
HRCT pattern: Non-fibrotic	44	775 [385.5–1219]	0.724
HRCT pattern: Fibrotic	62	784.5 [498–1168.5]	

Data are presented as median [interquartile range]. Comparisons between groups were performed using the Mann–Whitney U test. Two-sided *p*-values are reported. DLCO, Hemoglobin-corrected diffusing capacity of the lung for carbon monoxide; FVC, forced vital capacity; HRCT, high-resolution computed tomography.

**Table 3:** Comparison of clinical characteristics according to serum KL-6 category (cutoff 500 U/mL).

Variable		KL-6 < 500 (*n* = 35)	KL-6 ≥ 500 (*n* = 77)	*p*
Age, years		67 [57–75.5]	72 [65–77]	0.029
BMI, kg/m^2^		24.2 [22.7–27.8]	26.4 [24.2–28.8]	0.033
Female sex		29/35 (82.9%)	52/77 (67.5%)	0.093
Current/former smoking history		11/35 (31.4%)	38/77 (49.4%)	0.076
Cough		4/35 (11.4%)	13/76 (17.1%)	0.440
Dyspnea		15/35 (42.9%)	54/76 (71.1%)	0.004
Underlying immune-mediated disease				0.893
	Systemic sclerosis	11/35 (31.4%)	23/77 (29.9%)	
	Rheumatoid arthritis	6/35 (17.1%)	23/77 (29.9%)	
	Inflammatory myopathy	7/35 (20.0%)	9/77 (11.7%)	
	IPAF	2/35 (5.7%)	4/77 (5.2%)	
	Overlap syndrome	2/35 (5.7%)	4/77 (5.2%)	
	Sjögren’s disease	2/35 (5.7%)	4/77 (5.2%)	
	Sarcoidosis	2/35 (5.7%)	3/77 (3.9%)	
	Other diagnoses	3/35 (8.6%)	7/77 (9.1%)	
Duration of immune-mediated disease, years		8 [6–20]	7 [3–14]	0.067
Duration of ILD, years		8 [1–14]	4 [2–7]	0.056
Treatment				
	Corticosteroids	16/35 (45.7%)	39/77 (50.6%)	0.628
	Mycophenolate/Azathioprine	13/35 (37.1%)	25/77 (32.5%)	0.628
	Rituximab	3/35 (8.6%)	15/77 (19.5%)	0.145
	Hydroxychloroquine	8/35 (22.9%)	4/77 (5.2%)	0.005
Antifibrotic treatment		1/35 (2.9%)	19/77 (24.7%)	0.005
Autoantibodies				
	ANA positive	23/35 (65.7%)	52/73 (71.2%)	0.560
	Anti-CCP positive	6/20 (30.0%)	14/57 (24.6%)	0.633
	Rheumatoid factor, IU/mL	9.9 [7–18.4]	10 [9.1–50.1]	0.072
Systemic inflammatory biomarkers				
	CPK, U/L	75 [64–106]	73.5 [53.5–114]	0.614
	CRP, mg/L	3.0 [1.3–6]	4.9 [1.1–12.2]	0.205
HRCT pattern: Fibrotic		15/31 (48.4%)	47/75 (62.7%)	0.175
Pulmonary function tests				
	FVC, L	2.71 [2.22–3.26]	2.38 [1.96–2.92]	0.026
	FVC, % predicted	91 [84–99]	82 [69.5–94.8]	0.013
	FVC z-score	−0.53 [−1.1–−0.04]	−1.11 [−1.9–−0.41]	0.015
	FEV_1_, L	2.02 [1.76–2.49]	1.87 [1.61–2.20]	0.061
	FEV_1_, % predicted	91 [83–98]	84.5 [73.3–97]	0.091
	FEV_1_ z-score	−0.57 [−1.05–−0.22]	−1.03 [−1.62–−0.23]	0.197
	FEV_1_/FVC ratio	0.78 [0.73–0.82]	0.81 [0.75–0.86]	0.044
	DLCO, % predicted	77 [61–88]	61 [47.8–72]	< 0.001
	DLCO z-score	−1.49 [−2.51–−0.71]	−2.33 [−3.01–−1.63]	0.001

Data are presented as median [interquartile range] for continuous variables and as *n*/*N* (%) for categorical variables, where *N* represents the number of patients with available data within each KL-6 category. Percentages were calculated by column. Comparisons between groups were performed using the Mann–Whitney U test for continuous variables and the chi-square test or Fisher’s exact test, as appropriate. Missing values were not imputed, and analyses were conducted using available cases. Two-sided *p*-values are reported. ANA, antinuclear antibodies; anti-CCP, anti-cyclic citrullinated peptide; BMI, body mass index; CPK, creatine phosphokinase; CRP, C-reactive protein; DLCO, hemoglobin-corrected diffusing capacity of the lung for carbon monoxide; FEV_1_, forced expiratory volume in 1 s; FVC, forced vital capacity; HRCT, high-resolution computed tomography; ILD, interstitial lung disease; IPAF, interstitial pneumonia with autoimmune features. The number of available observations varied across variables because of the retrospective study design. Available observations for continuous variables were as follows: age, *n* = 110; BMI, *n* = 112; duration of immune-mediated disease, *n* = 108; duration of ILD, *n* = 106; rheumatoid factor, *n* = 103; CPK, *n* = 91; CRP, *n* = 110; FVC and FEV_1_ absolute and % predicted values, *n* = 111; FVC and FEV_1_ z-scores, *n* = 108; FEV_1_/FVC ratio, *n* = 111; DLCO % predicted, *n* = 109; and DLCO z-score, *n* = 108. Variable-specific denominators for categorical variables, including autoantibodies and HRCT classification, are reported directly in the table.

## Data Availability

The data analyzed in this study are not publicly available because they contain clinical information subject to ethical and data protection restrictions.

## Abbreviations

The following abbreviations are used in this manuscript:

ANA	Antinuclear antibodies
anti-Ro	Anti–Ro/SSA antibodies
anti-Scl70	Anti–topoisomerase I
BMI	Body mass index
CPK	Creatine phosphokinase
CRP	C-reactive protein
DLCO	Hemoglobin-corrected diffusing capacity of the lung for carbon monoxide
FEV_1_	Forced expiratory volume in 1 s
FVC	Forced vital capacity
GLI	Global Lung Function Initiative
HRCT	High-resolution computed tomography
ILD	Interstitial lung disease
IPAF	Interstitial pneumonia with autoimmune features
KL-6	Krebs von den Lungen-6
mMRC	Modified Medical Research Council
OR	Odds ratios
